# Heparan sulfate assists SARS-CoV-2 in cell entry and can be targeted by approved drugs in vitro

**DOI:** 10.1038/s41421-020-00222-5

**Published:** 2020-11-04

**Authors:** Qi Zhang, Catherine Zhengzheng Chen, Manju Swaroop, Miao Xu, Lihui Wang, Juhyung Lee, Amy Qiu Wang, Manisha Pradhan, Natalie Hagen, Lu Chen, Min Shen, Zhiji Luo, Xin Xu, Yue Xu, Wenwei Huang, Wei Zheng, Yihong Ye

**Affiliations:** 1 Laboratory of Molecular Biology, National Institute of Diabetes and Digestive and Kidney Diseases, National Institutes of Health, Bethesda, MD 20892 USA; 2 National Center for Advancing Translational Sciences, National Institutes of Health, Rockville, MD 20850 USA

**Keywords:** Glycobiology, Endocytosis

## Abstract

The cell entry of SARS-CoV-2 has emerged as an attractive drug repurposing target for COVID-19. Here we combine genetics and chemical perturbation to demonstrate that ACE2-mediated entry of SARS-Cov and CoV-2 requires the cell surface heparan sulfate (HS) as an assisting cofactor: ablation of genes involved in HS biosynthesis or incubating cells with a HS mimetic both inhibit Spike-mediated viral entry. We show that heparin/HS binds to Spike directly, and facilitates the attachment of Spike-bearing viral particles to the cell surface to promote viral entry. We screened approved drugs and identified two classes of inhibitors that act via distinct mechanisms to target this entry pathway. Among the drugs characterized, Mitoxantrone is a potent HS inhibitor, while Sunitinib and BNTX disrupt the actin network to indirectly abrogate HS-assisted viral entry. We further show that drugs of the two classes can be combined to generate a synergized activity against SARS-CoV-2-induced cytopathic effect. Altogether, our study establishes HS as an attachment factor that assists SARS coronavirus cell entry and reveals drugs capable of targeting this important step in the viral life cycle.

## Introduction

The ongoing pandemic of the coronavirus disease 2019 (COVID-19) has claimed many lives and severely damaged the global economy. This severe acute respiratory syndrome (SARS) is caused by a novel coronavirus SARS-CoV-2^1,2^, which is closely related to SARS-Cov, the virus underlying the 2003 SARS outbreak^3^.

As a positive-sense, single-stranded RNA virus bearing a membrane envelope, coronavirus enters cells when this membrane envelope fuses with host membranes^4^. Viral entry may take place either at the plasma membrane or at the endosomes following receptor-mediated endocytosis^5^. Previous studies showed that SARS-Cov enters cells primarily through ACE2-dependent endocytosis^5–9^. New evidence suggests that SARS-CoV-2 may follow the same entry path^1,10,11^. Specifically, limiting the production of phosphatidylinositol 4,5-bisphosphate (PIP2) inhibits the fusion of the SARS-CoV-2 envelop with the endolysosomes and viral entry^12,13^. Additionally, viral entry-associated membrane fusion requires a priming step mediated by several host proteases including the lysosome-localized Cathepsin B/L and serine proteases of the TMPRSS family^12,14–18^. Although some TMPRSS proteases may act on the cell surface, the fact that increasing the lysosomal pH in either TMPRSS2-positive Caco-2 or TMPRSS2-negative HEK293 cells both inhibits SARS-CoV-2 cell entry^14^ suggests endolysosomes as a major entry site for SARS-CoV-2 at least in certain cell types.

Our previous study on the intercellular transmission of misfolded α-Synuclein (α-Syn) fibrils, a cellular process reminiscent of viral infection, revealed an endocytosis mechanism by which the cell surface heparan sulfate (HS) facilitates receptor-mediated uptake of protein assemblies bearing excess positive charges^19^. HS is a negative charge-enriched linear polysaccharide molecule that is attached to several membrane and extracellular proteins collectively termed as heparan sulfate proteoglycans (HSPG). The cell surface HS can serve as an anchor to facilitate endocytosis of many cargos^20,21^, which include SARS-CoV-2-related coronaviruses^22–25^. To identify drugs that can diminish the spreading of misfolded α-Syn and the associated Parkinsonism^26^, we screen and identify eight FDA-approved drugs that block HS-dependent endocytosis of preformed α-Syn fibrils (see below). Intriguingly, one of the candidates Tilorone was recently reported as an inhibitor of SARS-CoV-2 infection^27^. This coincidence, together with the recently reported interaction of SARS-CoV-2 Spike with the HS mimetic glycan heparin^28–30^ and other evidence in support of a role for HS in the entry of SARS-CoV-2-related coronaviruses^22–25,31^ prompt us to investigate the possibility of targeting HS as a COVID-19 therapeutic strategy.

## Results

### Heparan sulfate facilitates Spike-dependent viral entry

To study Spike-mediated viral entry, we constructed luciferase-expressing pseudoviral particles (PP) bearing SARS-Cov or SARS-CoV-2 Spike. We infected HEK293T (human embryonic kidney cells) cells or HEK293T cells stably expressing ACE2-GFP with PP. As expected, ACE2-GFP HEK293T cells transduced with SARS-Cov or SARS-CoV-2 PP expressed luciferase significantly higher than noninfected ACE2-GFP cells (Supplementary Fig. S1a, b) or infected HEK293T cells without ACE2-GFP (Supplementary Fig. S1c). These results confirm Spike-dependent entry of SARS-Cov and CoV-2 via ACE2.

**Fig. 1:**
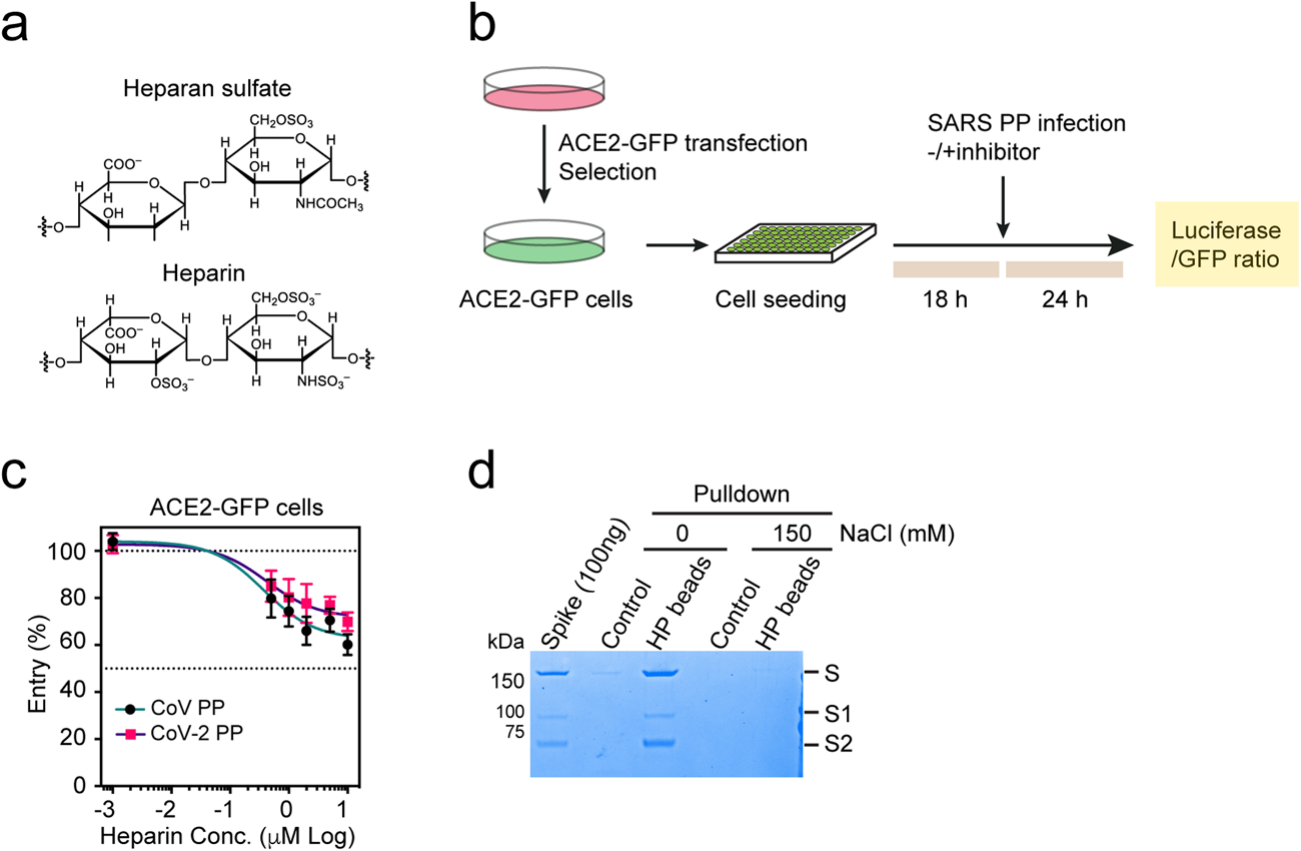
Heparin inhibits Spike-mediated SARS-Cov and CoV-2 entry. **a** The chemical structures of heparan sulfate and heparin. **b** The experimental scheme for inhibitor testing in HEK293T-ACE2-GFP cells. **c** Heparin mitigates the entry of SARS-Cov and SARS-CoV-2 PP. ACE2-GFP HEK293T cells were transduced with SARS-Cov and SARS-CoV-2 PP in the presence of heparin as indicated. The ratio of luciferase vs GFP was measured 24 h post-transduction. Error bars indicate SEM, *n* = 4. **d** Heparin interacts with Spike in a salt sensitive manner. Spike (600 ng) was incubated with either control or heparin-conjugated beads in a buffer containing 0 or 150 mM NaCl. Bound proteins were analyzed by SDS-PAGE and Coomassie blue staining. Note that a small amount of Spike interacts with heparin in the presence of 150 mM NaCl.

To test the role of HS in viral entry, we first treated ACE2-GFP HEK293T cells with heparin, a HS mimetic glycan frequently used as a competitive inhibitor for HS (Fig. 1a)^32,33^. Heparin treatment dose-dependently mitigated luciferase expression from both SARS-Cov and CoV-2 PP infection (Fig. 1b, c) with little impact on cell viability (Supplementary Fig. S1d). Heparin treatment also reduced luciferase expression in SARS-Cov- or CoV-2-transduced human lung epithelial Calu-3 cells (Supplementary Fig. S1e) and the inhibition was more significant than that in ACE2-GFP-overexpressing cells. Heparin pulldown showed that the purified Spike ectodomain readily bound to heparin-conjugated beads (Fig. 1d). As expected, the interaction was sensitive to salt, but a significant amount of Spike remained associated with heparin in a buffer containing 120 mM NaCl (Supplementary Fig. S1f), a condition recapitulating the airway surface salt concentration^34^. These data suggest that heparin acts as a competitive inhibitor for Spike-mediated viral entry and this inhibitory activity could be in part offset by overexpression of ACE2.

**Fig. 2:**
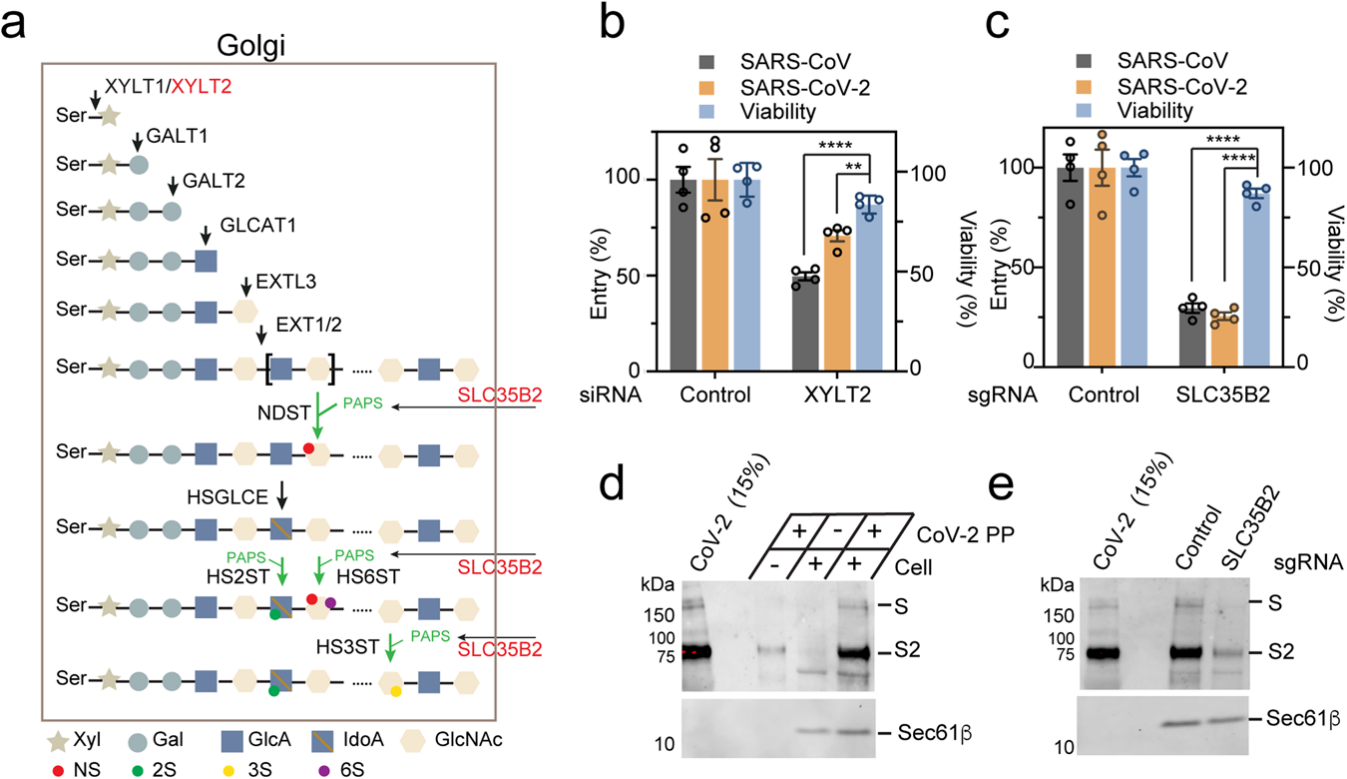
Heparan sulfate promotes Spike-mediated SARS-Cov and CoV-2 entry. **a** The HSPG biosynthetic pathway. Genes chosen for knockdown or knockout (KO) are in red. **b** Knockdown of *XYLT2* reduces SARS-Cov and SARS-CoV-2 PP entry. ACE2-GFP cells transfected with either control or *XYLT2* siRNA were transduced with SARS-Cov (gray) or SARS-CoV-2 (orange) PP for 24 h and the ratio of luciferase/GFP was determined. A parallel experiment done without the virus provides another control for the effect of gene knockdown on cell viability (blue). Error bars indicate SEM, *n* = 4, **P < 0.01, ****P < 0.0001 by unpaired Student *t*-test. **c** *SCL35B2* is required for SARS-Cov and SARS-CoV-2 cell entry. As in **b**, except that control and *SLC35B2* CRISPR KO cells were used. **d**, **e** SLC35B2 promotes the binding of SARS-CoV-2 PP to cells. **d** ACE2-GFP cells were spin-infected at 4 °C for 1 h. After washing, the virus bound to the cells was detected by immunoblotting. **e** The binding of SARS-CoV-2 PP to control and *SLC35B2*-deficient cells was analyzed by immunoblotting. S and S2 indicate the full-length and furin-cleaved S2 fragment, respectively.

Next, we used small interfering RNA (siRNA) or CRISPR to disrupt genes encoding HSPG biosynthetic enzyme XYLT2 and SLC35B2^19^ (Fig. 2a; Supplementary Fig. S1g–i). *XYLT2* encodes one of the two HS chain initiation enzymes. SLC35B2 is a Golgi-localized transporter for 3’-phosphoadenosine 5’-phosphosulfate (PAPS), which is essential for HS chain sulfation^32^. Knockdown of *XLYT2* by ~80% inhibited SARS-Cov and SARS-CoV-2 PP entry similarly as heparin treatment (Fig. 2b). By contrast, CRISPR-mediated inactivation of *SLC35B2* completely abolished HSPG biosynthesis^19^ and inhibited the entry of SARS-Cov and SARS-CoV-2 more dramatically (Fig. 2c). Analyses of GFP fluorescence showed no effect of *SCL35B2* knockout on ACE2-GFP expression (Supplementary Fig. S1j). Nevertheless, the knockout of *SLC35B2* significantly reduced the binding of SARS-CoV-2 PP to ACE2-GFP cells (Fig. 2d, e). Altogether, these results support a model in which the cell surface HS serves as a virus-recruiting factor to promote ACE2-dependent viral entry.

### A drug screen identifies inhibitors targeting the HS-dependent cell entry pathway

**Fig. 3:**
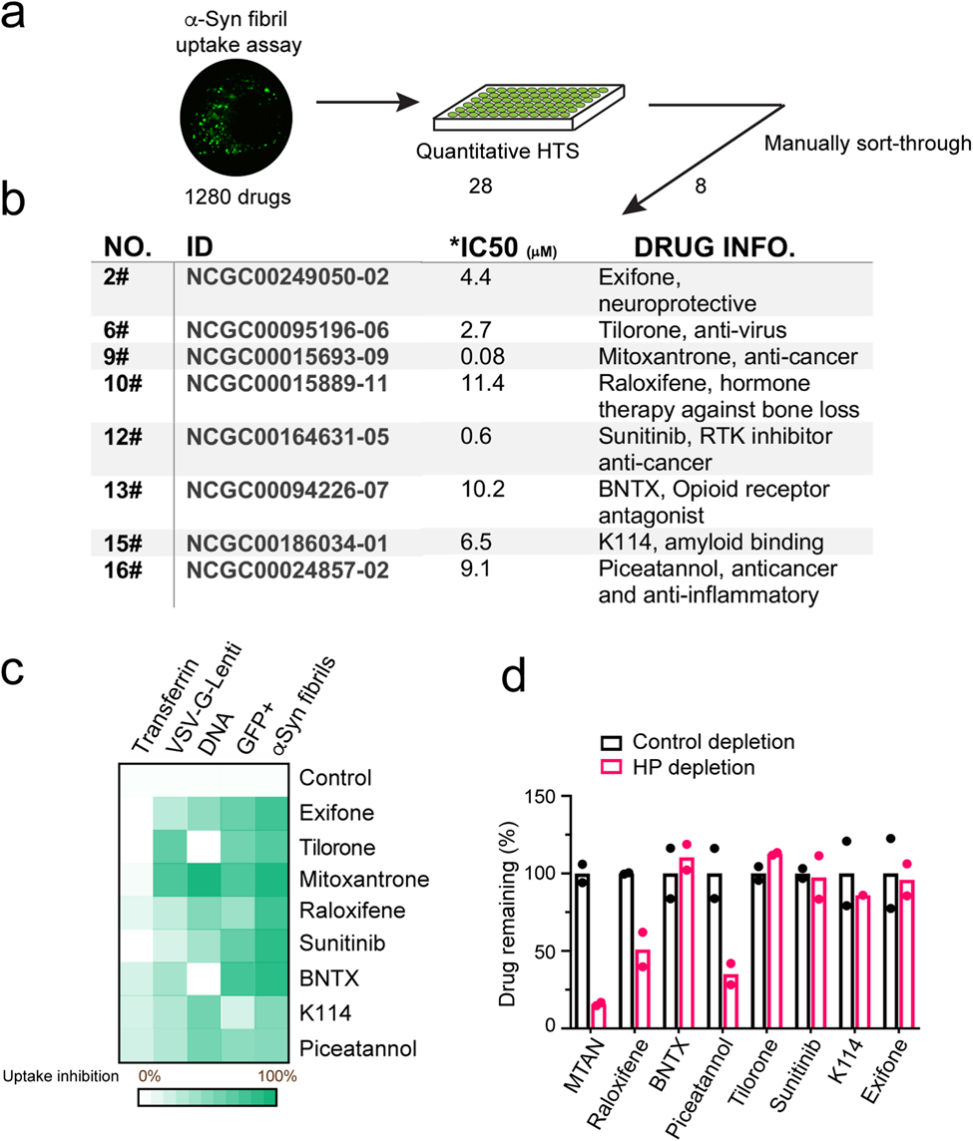
Two classes of drugs targeting HS-dependent cargo entry. **a** A high-throughput drug screen for inhibitors that block the uptake of fluorescently labeled α-Syn fibrils. **b** A summary of the drugs identified. IC_50_ was determined by a titration experiment. **c** A heatmap summary of the endocytosis inhibitory activity for the indicated drugs. All drugs were tested at 10 μM except for Sunitinib and Mitoxantrone (5 μM each). See Supplementary Fig. S2 for details. **d** Mass spectrometry analysis of drug binding to heparin beads. The indicated drugs were incubated with either control or heparin-conjugated beads. Unbound drugs were quantified by mass spectrometry. *n* = 2. MTAN, Mitoxantrone.

HS is a negatively charged biopolymer, which can recruit ligands bearing positive charges to facilitate their endocytosis^20^. We recently characterized the endocytosis mechanism of α-Syn fibril, which is a HS ligand^19^. We conducted a quantitative high-throughput screen (qHTS) using α-Syn filamentous inclusions to search for approved drugs that could block HS-dependent endocytosis (Fig. 3a). The screen identified 8 drugs that inhibited α-Syn fibril uptake in HEK293T cells (Fig. 3b). Additional studies using a panel of endocytosis cargos confirmed that the identified drugs are endocytosis inhibitors with a preference for HS-dependent ligands (Fig. 3c). Among the cargos tested, supercharged GFP (GFP^+^), polycation-coated DNA, and VSV-G-pseudotyped lentivirus all enter cells via a SLC35B2-dependent mechanism^19,35^, whereas transferrin uses a HS-independent but clathrin-dependent endocytosis pathway. The cholera toxin B chain (CTB) reaches the Golgi apparatus via a clathrin-independent mechanism^36^. None of the drugs tested affected the internalization of CTB, but Sunitinib and BNTX appeared to disrupt the perinuclear stacking of the Golgi system (Supplementary Fig. S2a, b). These drugs also had little impact on transferrin endocytosis (Supplementary Fig. S2c), but they could reduce the uptake of HS-dependent cargos to various levels with Mitoxantrone being the most potent one: at 5 μM, it almost completely blocked the endocytosis of all HS-dependent cargos tested (Fig. 3c; Supplementary Fig. S2d–f).

Biochemical binding coupled to mass spectrometry analyses showed that these drugs could be categorized into two classes depending on whether they bound to heparin. While Exifone, K114, Tilorone, BNTX, and Sunitinib showed no affinity to heparin, Mitoxantrone, Raloxifene, and Piceatannol in solution could be effectively depleted by heparin beads (Fig. 3d), suggesting that these drugs may target the cell surface HS directly to inhibit cargo uptake.

Among the drugs identified, Tilorone was previously established as a pan-antiviral agent^37^ that also inhibits SARS-CoV-2 infection in vitro^27^. This coincidence, together with the convergence of SARS-Cov and CoV-2 entry and α-Syn endocytosis at the HS glycans raise the possibility that inhibitors of HS-dependent endocytosis might also block SARS-Cov and CoV-2 entry. Indeed, with the exception of Exifone and K114, other drugs could all mitigate Spike-mediated viral entry in ACE2-GFP cells (Supplementary Fig. S3a and see below). Exifone and K114 were excluded from further study.

### The actin network is required for Spike-mediated viral entry

**Fig. 4:**
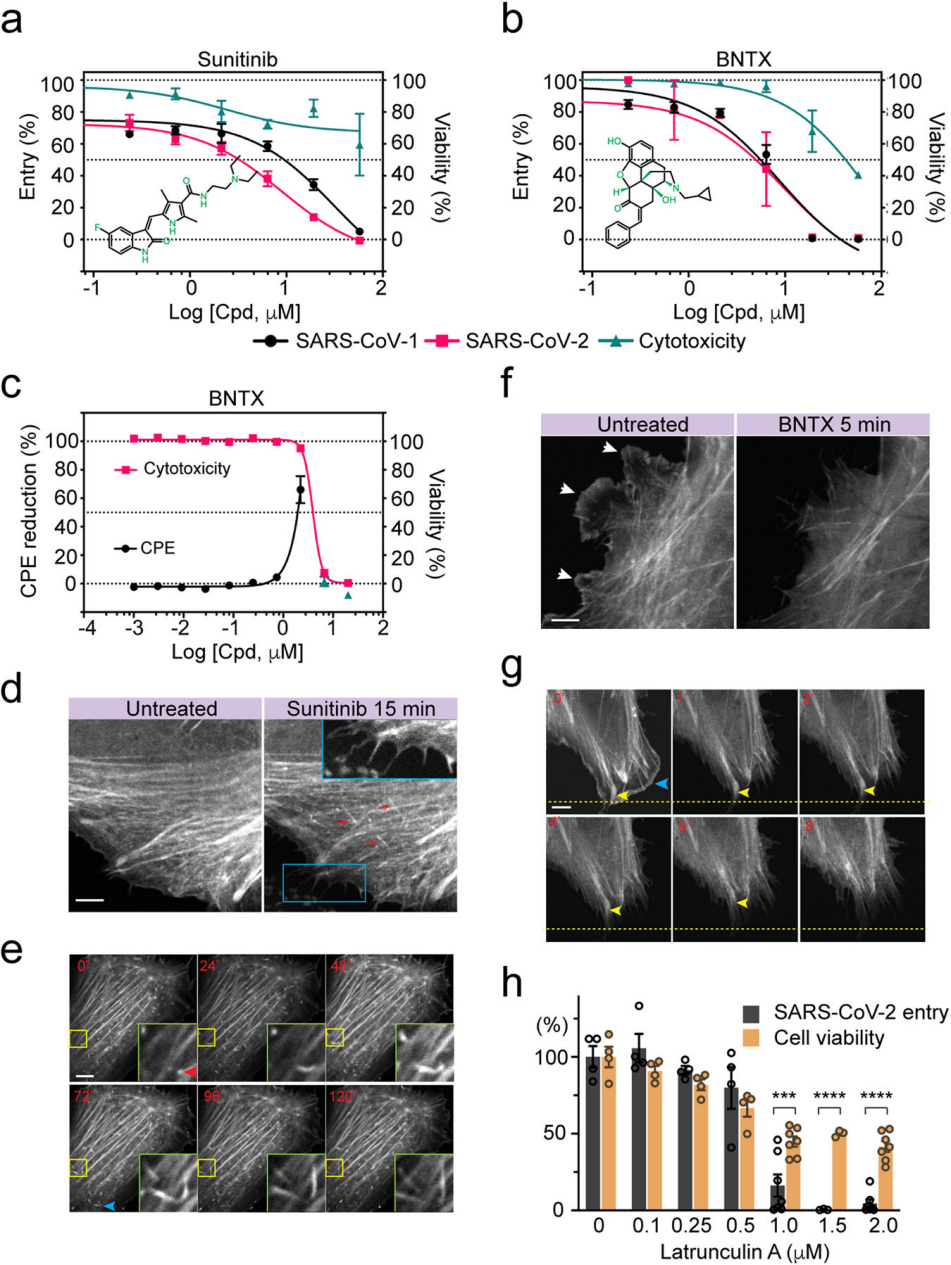
The actin cytoskeleton is required for HS-assisted viral entry. **a**, **b** Sunitinib and BNTX inhibit Spike-dependent entry of PP. HEK293-ACE2-GFP cells were transduced with SARS-Cov and SARS-CoV-2 PP in the presence the indicated drugs. The luciferase expression was measured 48 h post-transduction. Error bars indicate SEM, *n* = 4. As a control for cytotoxicity, cells treated with the drugs without virus were analyzed in parallel by an ATP-based cytotoxicity assay. **c** BNTX protects Vero E6 cells from SARS-CoV-2-induced CPE. Viability of Vero E6 cells was measured after treatment with the indicated drugs in the presence (black) or absence (red) of the SARS-CoV-2 virus. Error bars indicate SEM, *n* = 2. **d**, **e** Sunitinib stimulates actin filament formation at the cell periphery. **d** Confocal snapshots of a U2OS cell stably expressing Tractin-EGFP before or after Sunitinib (5 μM) treatment. The red arrows indicate new actin filaments formed in a direction perpendicular to the existing filaments. The inset shows an enlarged view of the box, which highlights newly formed filopodia. **e** Live-cell imaging of actin filament formation after Sunitinib treatment (5 μM, 60 min). The insets show an enlarged view of the boxed area. The red arrowhead in the inset and the blue arrowhead indicate two examples of actin filament assembly. Scale bar, 5 μm. **f**, **g** BNTX disrupts actin filaments at the cell periphery. **f** Confocal snapshots of a Tractin-EGFP cell before and after BNTX (10 μM) treatment. Arrows indicate the peripheral actin network disrupted by BNTX. **g** Live-cell imaging shows the shrinking of peripheral membranes after BNTX treatment. Yellow arrowheads mark a retracting actin bundle. The blue arrowhead indicates the cell edge. **h** Latrunculin A inhibits SARS-CoV-2 entry. ACE2-GFP cells incubated with SARS-CoV-2 PP in the presence of Latrunculin A were analyzed for luciferase expression (entry). A parallel treatment in the absence of the virus showed the toxicity of Latrunculin A (orange). Error bars indicate SEM. *n* = 2, ***P < 0.001, ****P < 0.0001.

We chose representative drugs of the two classes for further characterization. For those that did not bind heparin, we characterized BNTX and Sunitinib because their effect on Golgi morphology suggested a potential shared mechanism. BNTX is an opioid receptor antagonist for opioid or alcohol use disorders, while Sunitinib has been used as a receptor tyrosine kinase inhibitor for cancer therapy^38^. In HEK293-ACE2-GFP cells treated with SARS-Cov or SARS-CoV-2 PP, these drugs inhibited viral entry with respective IC_50_ of ~30 μM and 10 μM for Sunitinib, 9 μM and 10 μM for BNTX (Fig. 4a, b). In Vero E6 cells treated with wild-type SARS-CoV-2, BNTX at 2 μM rescued the virus-induced cytopathic effect (CPE) by ~70%, whereas 10 μM Sunitinib rescued the CPE only by ~30% (Fig. 4c; Supplementary Fig. S3b).

Because HS-assisted endocytosis often requires the actin cytoskeleton^19^, we determined whether these drugs affect the actin cytoskeleton network using U2OS cells stably expressing Tractin-EGFP, an actin filament label^39^. Confocal microscopy analysis showed that actin monomers polymerize near peripheral membranes in untreated cells, forming a microfilament network with parallel actin filaments (Supplementary Fig. S3c). Additionally, a meshwork of actin is associated with the cell cortex, while thick actin bundles named stress fibers are often found near the basal membranes. In cells exposed to Sunitinib, the actin microfilaments were frequently replaced by short disoriented actin segments. Many cells also contained an increased number of filopodia. By contrast, cells treated with BNTX had significantly reduced number of actin filaments, with many cells containing actin-positive aggregates (Supplementary Fig. S3c, top panels). These phenotypes were further confirmed by TIRF microscopy in Sunitinib- or BNT-treated cells (Supplementary Fig. S3d).

To further elucidate the actions of Sunitinib and BNTX, we used live-cell imaging to monitor the acute changes in actin dynamics in drug-treated cells. Shortly after Sunitinib treatment, we observed extensive de novo actin filament formation (Fig. 4d, e; Supplementary Movies S2 vs S1). Actin polymerization was also detected in the cell periphery where it drove filopodium formation (Fig. 4d) but was not seen near the basal membranes where stress fibers were located (Supplementary Movies S4 vs S3). Unlike Sunitinib, BNTX treatment resulted in rapid shrinking of actin microfilaments and the loss of membrane ruffling in the peripheral cortex (Fig. 4f, g; Supplementary Movies S6 vs S5). These data suggest that Sunitinib promotes actin assembly to form disoriented filaments, whereas BNTX disrupts the cortex actin meshwork either by promoting actin disassembly or inhibiting actin assembly. The fact that two structurally unrelated actin inhibitors both block SARS-Cov and CoV-2 entry strongly suggests that the entry of these viruses may require the actin cytoskeleton. Indeed, SARS-CoV-2 PP entry was also inhibited by Latrunculin A, a well-established actin inhibitor (Fig. 4h). These findings, together with the fact that Sunitinib was previously identified as an entry inhibitor for Ebola virus^40^ highlight an important role played by actin in the entry of these RNA envelop viruses.

### Mitoxantrone inhibits viral entry by targeting HS

**Fig. 5:**
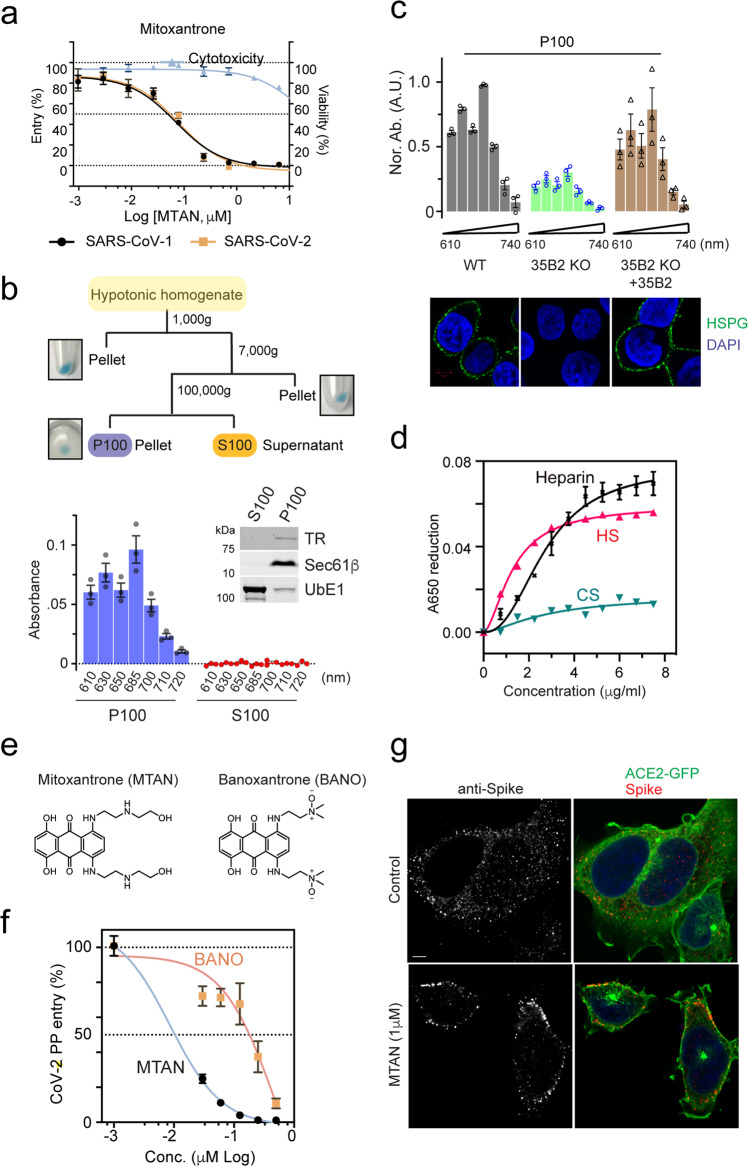
Mitoxantrone targets HS directly to inhibit SARS coronavirus entry. **a** Mitoxantrone inhibits Spike-mediated entry of PP. ACE2-GFP HEK293 cells were transduced with SARS-Cov and SARS-CoV-2 PP for 48 h in the presence of Mitoxantrone as indicated. Error bars indicate SEM, *n* = 4. Cells treated without the virus were used to control drug cytotoxicity. **b** The subcellular distribution of Mitoxantrone. The scheme illustrates the experimental procedure. HEK293T cells were treated with 5 μM Mitoxantrone for 30 min at 37 °C before fractionation. The membrane pellet (P100) and the cytosol supernatant (S100) fractions were analyzed by immunoblotting and by spectrometry at the indicated wavelengths. Error bars indicate SEM, *n* = 3. **c** The membrane association of Mitoxantrone requires *SLC35B2* (*35B2*). Cells of the indicated genotypes were treated with 5 μM Mitoxantrone for 30 min at 37 °C and then fractionated as in **b**. The absorbance of the P100 fractions was measured and the control sample at 680 nm was normalized to 1. Bottom panels show the cells stained with DAPI (blue) and GFP^+^ (green) to label DNA and HS (Green), respectively. Error bars indicate SEM, *n* = 3. **d** Mitoxantrone has a higher affinity for heparin and HS than chondroitin sulfate (CS). A_650_ of Mitoxantrone (5 μM) mixed with the indicated oligosaccharides was determined. The decrease in absorbance after the addition of the oligosaccharides was plotted. Error bars indicate SEM, *n* = 2. **e** The chemical structures of Mitoxantrone (MTAN) and Banoxantrone (BANO). **f** Banoxantrone (BANO) has reduced antiviral activity. ACE2-GFP HEK293T cells were incubated with SARS-CoV-2 in the presence of the indicated drugs for 24 h before measuring the luciferase activity. Error bars indicate SEM, *n* = 4. **g** Mitoxantrone inhibits SARS-CoV-2 cell entry. ACE2-GFP HEK293T cells were pretreated with DMSO as a control or Mitoxantrone for 30 min before incubation with SARS-CoV-2 PP. Cells were fixed 3 h later and stained with anti-Spike antibodies (red) and DAPI (blue).

Among drugs bound to heparin, Mitoxantrone had a higher affinity. Mitoxantrone was previously reported as a DNA intercalator, which also inhibits type II DNA topoisomerase^41,42^. Accordingly, Mitoxantrone is currently approved for treatment of acute nonlymphocytic leukemia, prostate cancer, and multiple sclerosis. In PP-treated ACE2-GFP HEK293 cells, Mitoxantrone strongly inhibited viral entry with IC_50_ > 100-fold lower than that of cytotoxicity, suggesting an excellent antiviral therapeutic window (Fig. 5a).

Because Mitoxantrone has absorbance peaks at 620 nm and 685 nm (Supplementary Fig. S4a), we used this spectral property to trace its localization in drug-treated cells, which hinted at its cellular target. To this end, we performed subcellular fractionation using Mitoxantrone-treated cells to obtain the nucleus (1000× *g* pellet), mitochondria-enriched heavy membrane (7000× *g* pellet), light membrane (100,000× *g* pellet), and cytosol (100,000× *g* supernatant) fractions (Fig. 5b, top panel). Although blue color was seen in every pellet fraction, Mitoxantrone in the nucleus and heavy membrane fractions was resistant to extraction by a buffer containing the non-ionic detergent NP40 or 1% SDS, probably due to tight association with DNA. By contrast, Mitoxantrone in the light membranes (containing the plasma membrane and endoplasmic reticulum as demonstrated by immunoblotting; Fig. 5b, middle panel) could be readily released by an NP40-containing buffer and detected by a spectrometer (Fig. 5b). No Mitoxantrone was detected in the cytosol fraction. Thus, in addition to DNA, Mitoxantrone also binds to cell membranes.

Several lines of evidence suggest that Mitoxantrone targets the cell surface HS directly. First, the amount of P100-associated Mitoxantrone from *SLC35B2*-deficient cells (lacking HS) was significantly lower than that of wild-type cells, which could be rescued by re-expression of SLC35B2 (Fig. 5c). Additionally, as mentioned above, Mitoxantrone readily bound to heparin, a glycan structurally related to HS (Fig. 3d; Supplementary Fig. S4b). Because the interaction of heparin with Mitoxantrone lowered its absorption spectrum in solution (Supplementary Fig. S4c), we used this property to further determine the interaction of Mitoxantrone with other related glycans. As expected, HS bound to Mitoxantrone similarly as heparin, but chondroitin sulfate (CS) did not bind to Mitoxantrone significantly (Fig. 5d). Moreover, Banoxantrone, an anticancer drug structurally related to Mitoxantrone did not show significant interaction with either heparin or HS (Fig. 5e; Supplementary Fig. S4d, e). These results suggest a specific and direct interaction between Mitoxantrone and HS, which explains the SLC35B2-dependent association of Mitoxantrone with the cell membranes. Importantly, compared to Mitoxantrone, Banoxantrone was a much weaker SARS-CoV-2 PP entry inhibitor (Fig. 5f), suggesting a crucial role for heparin/HS binding in the antiviral activity of Mitoxantrone.

Although Mitoxantrone binds HS directly, it did not disrupt the binding of SARS-CoV-2 PP to ACE2-GFP-expressing cells. Instead, when cells pretreated with Mitoxantrone were exposed to SARS-CoV-2 PP, most viral particles remained bound to the plasma membrane as revealed by immunostaining with an anti-Spike antibody (Fig. 5g). By contrast, in control cells, Spike antibodies detected mostly intracellular speckles, probably representing endocytosed viral particles (Fig. 5g). This result suggests that Mitoxantrone and Spike bind HS in different modes, but the binding of Mitoxantrone to HS alters its function to block viral entry.

Surprisingly, while weaker inhibitors like BNTX rescued the CPE of SARS-CoV-2 in Vero E6 cells, Mitoxantrone did not show any activity in this assay. This might be due to cytotoxicity linked to the DNA-binding off-target property of Mitoxantrone. Consistent with this notion, Vero E6 cells were more sensitive to Mitoxantrone-induced cell death (Supplementary Fig. S4f vs Fig. 5a). In this regard, it is compelling that the dose-dependent toxicity profiles of Mitoxantrone and Banoxantrone were indistinguishable (Supplementary Fig. S4f), which suggests the possibility of generating Mitoxantrone derivatives with similar antiviral activities but reduced cytotoxicity.

### A combination regimen optimally targeting the HS-assisted viral entry

**Fig. 6:**
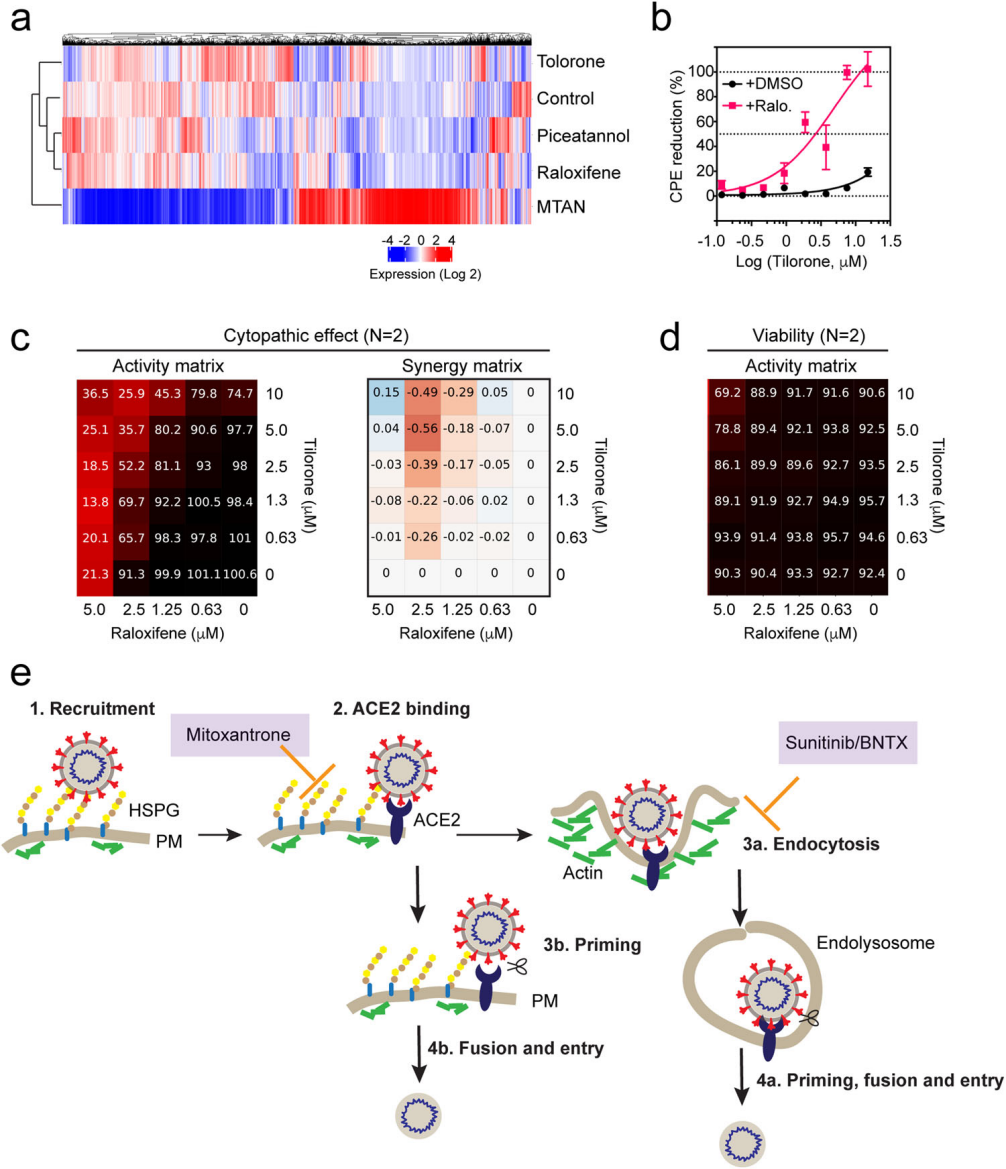
A combination regimen that optimally targets HS-dependent viral entry. **a** A heatmap showing the gene expression profiles of untreated HEK293T cells or cells treated with the indicated drugs at 10 μM for 6 h (Mitoxantrone 5 μM). **b**–**d** Drug combination studies show a synergistic CPE rescue effect by Tilorone and Raloxifene (Ralo.). **b** Vero E6 cells treated with Tilorone at different concentrations with (1.5 μM) or without Raloxifene were infected with SARS-CoV-2 for 72 h before viability test. Error bars indicate SEM. *n* = 2. **c**, **d** Matrix blocks for Tilorone plus Raloxifene in the CPE and cell viability assays. Activity blocks show normalized levels of viral toxicity (**c**) or normalized cell viability (no virus) (**d**). Red labels in the synergy matrix indicate positive synergism whereas blue labels suggest antagonism. **e** A SARS-CoV-2 entry map with the drug targeting sites identified in this study. PM, plasma membrane.

We next used gene expression profiling to further define the action of Tilorone, Raloxifene, and Piceatannol in comparison to Mitoxantrone. We treated HEK293T cells with these drugs at a dose that inhibited Spike-mediated viral entry. Differential gene expression analyses showed that Mitoxantrone had the most significant impact on gene expression, probably due to its strong DNA-binding activity (Fig. 6a). By contrast, Tilorone had the smallest effect, altering a limited number of genes by only small changes (Supplementary Fig. S5a–d). Cluster analysis showed that the gene expression signature associated with Raloxifene treatment was more similar to that of Piceatannol-treated cells than to Tilorone-treated cells (Fig. 6a), consistent with the observation that Raloxifene and Piceatannol but not Tilorone interact with heparin.

Conceptually, it might be possible to combine drugs targeting distinct steps in the HS-assisted entry pathway to produce a synergized antiviral activity. We tested the combination of Raloxifene with Tilorone because the latter has been used as an antiviral agent and because either Tilorone or Raloxifene by itself modestly rescued SARS-CoV-2-induced CPE in Vero E6 cells (Supplementary Fig. S5e, f). Strikingly, co-treatment with 1.5 μM Raloxifene reduced IC_50_ of Tilorone by ~10-fold in the CPE assay (Fig. 6b). The synergistic inhibition of viral CPE was further confirmed by a matrix test, in which Tilorone was tested systematically with different concentrations of Raloxifene (Fig. 6c). Importantly, no significant cytotoxicity was observed in combined treatment even at highest concentrations (Fig. 6d).

## Discussion

It is known that ACE2-mediated SARS-Cov and CoV-2 entry is controlled by a protease-dependent priming step^14–16^. Evidence presented in this study supports an additional layer of regulation imposed by the cell surface HS. The cell surface HS proteoglycans comprise of two protein families (6 Glypicans and 4 Syndecans)^32^. Proteins of these two families share no sequence similarity, but they all bear negatively charged HS polymers that can promote cell interactions with a variety of endocytic ligands^20,21^. Our data together with recent preprints in bioRxiv support a model in which HS glycans help to recruit SARS-CoV-2 to the cell surface, which increases its local concentration for effectively engaging ACE2^43,44^ (Fig. 6e). Interestingly, some other coronaviruses also use a similar two-receptor mechanism for cell entry. For example, some α-Coronaviruses first attach themselves to sialoglycans on the cell surface before binding to proteinaceous receptors^45,46^.

How do HS glycans interact with Spike? Two recent studies posted at bioRxiv modeled the interactions of heparin with Spike using either full-length Spike or just the RBD domain^29,44^. While several potential binding sites were suggested on full-length Spike, a more sensible model was generated when the RBD domain was used. This model suggests a long positive charge-enriched binding groove that accommodates a chain of heparin/HS. This model, if confirmed, is consistent with the observed salt sensitive heparin–Spike interaction.

How Mitoxantrone inhibits SARS-Cov and CoV-2 entry remains to be elucidated. It is known that Spike in the prefusion state adopts distinct conformations with the RBD domain either in the up or down position^44,47^, and the interaction of Spike with ACE2 requires the RBD-up conformation^11,48–50^. By contrast, the proposed HS-binding model is compatible with either the RBD-up or -down conformation^44^. Thus, it is possible that HS binding occurs on Spike with flexible RBD positions, and this conformational plasticity may facilitate downstream steps such as ACE2-dependent endocytosis and/or protease-mediated priming^51^. Mitoxantrone might influence the mode of HS interaction with the Spike and thus inhibits viral entry.

In addition to Mitoxantrone, we uncover several other drugs capable of targeting HS and HS-dependent endocytosis. The fact that many of these drugs also mitigate Spike-dependent viral entry strongly corroborates the idea that ACE2-mediated endocytosis serves a major role in SARS-CoV-2 cell entry. The identified drugs have a relatively limited impact on endocytosis as they affect neither clathrin-mediated transferrin uptake nor clathrin-independent uptake of CTB. Thus, these drugs are expected to be less toxic than previously reported pan-endocytosis inhibitors^13^. Among the drugs identified, Sunitinib and BNTX disrupt actin dynamics (Fig. 6e), suggesting the actin network as a previously unknown regulator for SARS-Cov and CoV-2 entry. These viruses may enter cells using receptor-mediated macropinocytosis, which depends on actin and has been known for its role in viral pathogen uptake^52^.

Although our study focuses on SARS-Cov and CoV-2, drugs targeting HS and HS-dependent endocytosis are expected to have a broad antiviral activity. Consistent with this view, Tilorone has long been recognized as a general antiviral agent. An early study in mice suggested that Tilorone administrated orally might upregulate interferon production^53^, but our gene expression study did not reveal significant changes in the expression of interferon by Tilorone treatment. Instead, the demonstration of Tilorone as an inhibitor of HS-dependent endocytosis offers an alternative explanation for the reported antiviral function. Importantly, our study also suggests that Raloxifene, a heparin/HS-binding drug can enhance the antiviral activity of Tilorone. Raloxifene is a selective estrogen receptor modulator used to treat osteoporosis and to prevent breast cancer in postmenopausal women (drugbank.ca/drugs/DB00481)^54^. Both Tilorone and Raloxifene are well tolerated and can be conveniently administrated orally. Thus, whether this combination can be clinically effective against SARS-CoV-2 infection deserves additional testing in animal and other antiviral models. In summary, our study not only establishes HS-dependent viral entry as a novel drug target for COVID-19, but also reveals candidate drugs that disrupt this critical process in the SARS-CoV-2 life cycle.

## Materials and methods

### Chemicals and reagents

The initial screening library LOPAC^R1280^ was purchased from Sigma. The chemicals for the follow-up studies were purchased as indicated in Supplementary Table S1. Standard quality control by HPLC was conducted before the drugs were used in the study. pcDNA3.1-SARS-CoV-2-Spike plasmid was obtained from BEI resource^11^. Other chemicals and reagents were listed in Supplementary Table S1.

### Cell line, transfection, lentivirus production, and infection

HEK293T cells stably expressing human ACE2 tagged with GFP (ACE2-GFP) were generated by transfecting cells with pCMV-ACE2-GFP (Covex), and stable clones were hand-picked after neomycin (1 mg/mL) selection for 1 week. Two ACE2-GFP cell lines using either HEK293 or HEK293T as the parental line were independently generated and used at NCATS and NIDDK, respectively. U2OS cells stably expressing Clathrin light chain-mCherry and Tractin-EGFP and the CRISPRv2 construct expressing sgRNA targeting *SLC35B2* were described previously^19^. Calu-3 cells were purchased from ATCC and maintained in MEM with 10% fetal bovine serum. sgRNA-expressing lentiviruses were produced by transfecting 1 million 293FT cells (Thermo Fisher Scientific) in a 3.5-cm dish with 0.4 µg pVSV-G, 0.6 µg psPAX2, and 0.8 µg CRISPRv2-sgRNA. Transfected cells were incubated with 3 mL fresh DMEM medium for 72 h before viruses were harvested.

### α-Syn fibrils uptake assay and drug screen

Fluorescence dye labeled α-Syn preformed fibrils were generated as previously described^19^. siRNA-mediated gene silencing was performed by lipofectamine RNAiMAX (Invitrogen) mediated transfection following the instruction from the manufacture. HEK293T cells were seeded in white 1536-well microplates that have transparent bottom (Greiner BioOne) at 2000 cells/well in 2 µL media and incubated at 37 °C overnight (~16 h). pHrodo red-labeled α-Syn fibrils were added at 400 nM final concentration to each well by a dispenser. After 1 h incubation, compounds from the LOPAC^R1280^ library (Sigma) were titrated 1:3 in DMSO and dispensed via pintool at 23 nL/well to the assay plates. After 24 h of incubation, the fluorescence intensity of pHrodo red was measured by a CLARIOstar Plus plate reader (BMG Labtech). Data were normalized using cells containing 400 nM pHrodo red-labeled Syn fibrils as 100% and medium containing 400 nM preformed fibrils-pHrodo red as 0%.

### SARS-Cov and SARS-CoV-2 PP

SARS-Cov-S, SARS-CoV-2-S, and ΔEnv (lack the Spike protein) PP were custom manufactured by the Codex Biosolutions (Gaithersburg, MD) following previously reported methods using a murine leukemia virus (MLV) pseudotyping system^55,56^. The SARS-CoV-2-S construct with Wuhan-Hu-1 sequence (BEI #NR-52420) was C-terminally truncated by 19 amino acids to reduce ER retention for pseudotyping^12^.

### PP entry assay in the 1536-well format

HEK293-ACE2 cells seeded in white, solid bottom 1536-well microplates (Greiner BioOne) at 2000 cells/well in 2 µL medium were incubated at 37 °C with 5% CO_2_ overnight (~16 h). Compounds were titrated 1:3 in DMSO and dispensed via pintool at 23 nL/well to the assay plates. Cells were incubated with compounds for 1 h at 37 °C with 5% CO_2_ before 2 µL/well of PP were added. The plates were then spinoculated by centrifugation at 1500 rpm (453× *g*) for 45 min and incubated for 48 h at 37 °C, 5% CO_2_ to allow cell entry of PP and the expression of luciferase. After the incubation, the supernatant was removed with gentle centrifugation using a Blue Washer (BlueCat Bio). Then 4 µL/well of Bright-Glo luciferase detection reagent (Promega) was added to assay plates and incubated for 5 min at room temperature. The luminescence signal was measured using a PHERAStar plate reader (BMG Labtech). Data were normalized with wells containing PP as 100% and wells containing control ΔEnv PP as 0%. Experiments shown in Fig. 4a, b, Supplementary Figs. S3a, S5a were conducted in this format at NCATS.

### PP entry assay in the 96-well format

HEK293T-ACE2-GFP cells were seeded in white, transparent bottom 96-well microplates (Thermo Fisher Scientific) at 20,000 cells per well in 100 µL growth medium and incubated at 37 °C with 5% CO_2_ overnight (~16 h). The growth medium was carefully removed and 50 µL PP or PP-containing compounds were added into each well. The plates were then spinoculated by centrifugation at 1500 rpm (453× *g*) for 45 min and incubated for 24 h (48 h for Calu-3 cells) at 37 °C, 5% CO_2_ to allow cell entry of PP and the expression of luciferase. After incubation, the supernatant was carefully removed. Then 50 µL/well of Bright-Glo luciferase detection reagent (Promega) was added to assay plates and incubated for 5 min at room temperature. The luminescence signal was measured by a Victor 1420 plate reader (PerkinElmer). For ACE2-GFP cells, the GFP signal was also determined by the plate reader. Data were normalized with wells containing PP but no compound as 100%, and wells mock-treated with phosphate buffer saline (PBS) as 0%, and the ratio of luciferase to the corresponding GFP intensity was calculated. Experiments shown in Figs. 1c, 2b, c, and 5f were done following this protocol at NIDDK.

### ATP content cytotoxicity assay in the 1536-well format

HEK293-ACE2 cells were seeded in white, solid bottom 1536-well microplates (Greiner BioOne) at 2000 cells/well in 2 µL medium and incubated at 37 °C with 5% CO_2_ overnight (~16 h). Compounds were titrated 1:3 in DMSO and dispensed via pintool at 23 nL/well to assay plates. Cells were incubated for 1 h at 37 °C, 5% CO_2_ before 2 µL/well of media was added. The plates were then incubated at 37 °C, 5% CO_2_ for 48 h. After incubation, 4 µL/well of ATPLite (PerkinElmer) was added to assay plates and incubated for 15 min at room temperature. The luminescence signal was measured using a Viewlux plate reader (PerkinElmer). Data were normalized with wells containing cells as 100%, and wells containing media-only as 0%.

### ATP content cytotoxicity assay in the 96-well format

HEK293T-ACE2-GFP cells were seeded in white, transparent bottom 96-well microplate (Thermo Fisher Scientific) at 20,000 cells per well in 100 µL/well growth medium and incubated at 37 °C with 5% CO_2_ overnight (~16 h). The growth medium was carefully removed and 100 µL medium with compounds was added into each well. The plates were then incubated at 37 °C for 24 h (48 h for Calu-3 cells) at 37 °C 5% CO_2_. After incubation, 50 µL/well of ATPLite (PerkinElmer) was added to assay plates and incubated for 15 min at room temperature. The luminescence signal was measured using a Victor plate reader (PerkinElmer). Data were normalized with wells containing cells but no compound as 100%, and wells containing media-only as 0 %.

### SARS-CoV-2 CPE assay

SARS-CoV-2 CPE assay was conducted at the Southern Research Institute (Birmingham, AL) as fee-for-service. Briefly, compounds were titrated in DMSO and acoustically dispensed into 384-well assay plates at 60 nL/well. Cell culture medium (MEM, 1% Pen/Strep/GlutaMax, 1% HEPES, 2% HI FBS) was dispensed at 5 µL/well into assay plates and incubated at room temperature. Vero E6 (selected for high ACE2 expression) was inoculated with SARS-CoV-2 (USA_WA1/2020) at 0.002 M.O.I. in media and quickly dispensed into assay plates at 4000 cells/well in 25 µL volume. Assay plates were incubated for 72 h at 37 °C, 5% CO_2_, 90% humidity. Then, 30 µL/well of CellTiter-Glo (Promega) was dispensed, incubated for 10 min at room temperature, and the luminescence signal was read on an EnVision plate reader (PerkinElmer). An ATP content cytotoxicity assay was conducted with the same protocol as CPE assay except that SARS-CoV-2 virus was omitted from the incubation.

### GFP^+^ purification and HSPG staining

His-tagged GFP^+^ was reported previously^19^. To stain the cell surface HSPG by GFP^+^, we incubated cells with 200 nM GPF^+^ in the growth medium on ice for 15 min. Cells were then fixed and stained with DAPI to reveal the nucleus.

### Endocytosis and viral binding assays

To test the effect of the compounds on DNA uptake, we seeded HEK293T cells previously infected with a YFP-expressing retrovirus at 0.2 × 10^6^/well in a poly-lysine D-coated 12-well plate. 24 h later, cells were treated with the chemicals for 30 min before a transfection mixture containing 0.2 µg mCherry-expressing plasmid and 0.6 µL TransIT293 (Mirus) was added to the cells. Cells were incubated with the DNA for 4 h, after which the medium was replenished. The cells were further grown for 48 h. Cells were then lysed in NP40 lysis buffer and the fluorescence intensity in cleared lysates was measured by a Fluoromax3 fluorometer (Horiba).

To test the effect of compounds on VSV-G-coated lentivirus uptake/infection, HEK293T cells expressing YFP were seeded at 0.2 × 10^6^/well in a poly-lysine D-coated 12-well plate. After 24 h, cells were treated with compounds for 30 min and then infected with a mCherry-expressing lentivirus at M.O.I. of ~2.0 in the presence of the compounds for 6 h. The virus and compounds were removed, and cells were further grown in fresh medium for 48 h before fluorescence measurement.

To test the effect of compounds on GFP^+^ or transferrin uptake, HEK293T cells were seeded at 25,000 cells per well in an 8-well chamber (ibidi). 24 h later, cells were treated with compounds for 30 min before the addition of GFP^+^ (200 nM) or transferrin (Thermo Fisher Scientific) (50 μg/mL). Cells were further incubated at 37 °C for 4 h before fixation and confocal imaging.

To detect the binding of SARS-CoV-2 to the cell surface, HEK293T-ACE2-GFP cells seeded in 24-well plates that had been coated with fibronectin were treated with 50 μL virus per well at 4 °C for 1 h with centrifugation at 1500 rpm (453× *g*) for 60 min. Cells were carefully washed with ice-cold PBS to remove unbound virus and then lysed in a NP40 lysis buffer containing 0.5% Igepal, 20 mM Tris, pH 7.4, 150 mM Sodium Chloride, 2 mM Magnesium Chloride, 0.5 mM EDTA, 1 mM DTT, and a protease inhibitor cocktail. The cell extracts cleared by centrifugation (16,000× *g*, 5 min) were analyzed by immunoblotting.

### UPLC-MS/MS assay

Ultra-performance liquid chromatography-tandem mass spectrometry (UPLC-MS/MS) methods were developed and optimized to determine compounds’ concentrations in the in vitro samples. Mass spectrometric analysis was performed on a Waters Xevo TQ-S triple quadrupole instrument using electrospray ionization in positive mode (Mitoxantrone, BNTX, Tilorone and Sunitinib) and negative mode (Raloxifene, Piceatannol, Exifone, K114) with the selected reaction monitoring. The separation of test compounds was performed on an Acquity BEH C_18_ column (50 × 2.1 mm, 1.7 µL) using a Waters Acquity UPLC system with 0.6 mL/min flow rate and 2 min gradient elution. The mobile phases were 0.1% formic acid in water and 0.1% formic acid in acetonitrile. The calibration standards (0.100–100 µM) and quality control samples were prepared in 50% acetonitrile/water with 0.1% formic acid and PBS buffer. Aliquots of 10 µL samples were mixed with 200 µL internal standard in acetonitrile to precipitate proteins in a 96-well plate. 0.5 µL supernatant was injected for the UPLC-MS/MS analysis. Data were analyzed using MassLynx V4.1 (Waters Corp., Milford, MA).

### qRT-PCR analysis of knockout or knockdown cells

Total RNA was extracted from 3 million HEK293T cells using TriPure reagent (Roche) and purified using RNeasy MinElute Cleanup Kit (Promega) following the standard protocols. The RNA concentration was measured by Nanodrop 2000 UV spectrophotometer, and 1 µg total RNA was converted to cDNA using the iScript Reverse Transcription Supermix (BioRad) system. 1 µL cDNA was used to perform qPCR using SsoAdvanced SYBR Green supermix kit (BioRad) on a CFX96 machine (BioRad). Data were analyzed using BioRad CFX Manager 3.0 software. GADPH was used as a reference gene for the quantification of gene expression levels. Primers used for qRT-PCR were listed in the previous study^19^. For the RNAseq study, cells were treated independently for 6 h with each drug three times and six untreated control samples were included. The treatment conditions are Mitoxantrone 5 µM, Tilorone 10 µM, Raloxifene 10 µM, Piceatannol 10 µM. RNA isolated from control or drug-treated cells (10 µg/sample) were processed by Novagene USA.

### Membrane fractionation and heparin Sepharose pulldown

To fractionate cells, ~15 million cells were treated with 5 µM Mitoxantrone or Banoxantrone for 30 min at 37 °C. Cells were harvested and washed with ice-cold PBS. Cells were then resuspended and incubated in 900 µL of a hypotonic buffer (50 mM HEPES, pH 7.3, 25 mM potassium acetate) containing a protease inhibitor cocktail. Cells were homogenized in a Dounce homogenizer with a tight pestle and then subject to differential centrifugation at 1000× *g* for 5 min, 7000× *g* for 10 min, and 100,000× *g* for 20 min. The P100 membrane pellet was resuspended in 20 µL NP40 lysis buffer containing 20 mM Tris, pH 7.4, 0.5% NP40, 150 mM Sodium Chloride, 2 mM Magnesium Chloride. The absorbance in the cleared membrane extract and the S100 fraction was measured by a NanoDrop 2000 spectrometer. Note that no absorbance was detected for the S100 fraction even when the samples were measured by a conventional spectrometer with a ×10 light path.

To determine the binding of Mitoxantrone to heparin-coated Sepharose, the compound was diluted to 50 µM in PBS and then incubated with PBS-washed heparin beads or as a control with Sepharose. After brief mixing, the beads were sedimented by centrifugation. The supernatant fractions were analyzed by NanoDrop 2000 for light absorption at the wavelengths specified in the figure legends.

The pulldown of Spike by heparin beads were done by incubating 600 ng Spike with pre-washed heparin Sepharose in 25 mM Tris, pH 7.3, 2 mM KCl, 1 mM MgCl_2_, 0.05% Igepal with or without 150 mM NaCl. To test the effect of Mitoxantrone on heparin–Spike interaction, 30 µL heparin beads were washed with PBS, treated with 200 µL Mitoxantrone (100 µM) for 5 min at room temperature or as a control remained untreated. Unbound Mitoxantrone was removed after centrifugation. The beads were then incubated with 600 ng purified Spike protein in 300 µL PBS containing 0.05% NP40 and 1 mM DTT. The reaction was incubated at room temperature for 30 min. The beads were washed two times with PBS and eluted with 1× Laemmli buffer for SDS-PAGE and immunoblotting analysis.

### Drug synergy analysis

CPE and Toxicity were normalized using independent control wells (DMSO ± virus) on each plate, so activity values were not strictly bounded between [0, 100]. For CPE assay, DMSO + virus was treated as the neutral control, whereas DMSO-only (no virus) served as the positive control. Then normalized CPE = 1 − (x – neutralCtrl)/(positiveCtrl – neutralCtrl) × 100%. For viability assay, DMSO-only was used as the neutral control and media-only wells (no cell) as the negative control. Normalized viability = (x – negativeCtrl)/(neutralCtrl – negativeCtrl) × 100%. Synergism and antagonism from a 6 × 6 block were evaluated using the highest single agent model (HSA). Given a dose combination A_conc1_ + B_conc2_,

HSA(A_conc1_ + B_conc2_) = activity(A_conc1_ + B_conc2_) – MIN[activity(A_conc1_), activity(B_conc2_)]

Therefore, we have

Synergism: HSA(*) < 0 (value from combination is lower than the best single agent)

Antagonism: HSA(*) > 0 (value from combination is greater than the best single agent)

Additivity: HSA(*) = 0 (value from combination equals to best single agent)

The overall HSA synergism (HSA_sum_) given a 6 × 6 block was calculated as the sum of HSA(A_conc*_ + B_conc*_) across the non-toxic dose combinations (defined as viability activity > 80). We used empirical cutoff HSA_sum_ < −100 to call a synergistic combination. The highest concentration of Raloxifene was excluded from the figure because it alone induced significant cytotoxicity.

### Image processing and statistical analyses

Confocal images were processed using the Zeiss Zen software. To measure fluorescence intensity, we used the Fiji software. Images were converted to individual channels and region of interest was drawn for measurement. Statistical analyses were performed using either Excel or GraphPad Prism 8. Data are presented as means ± SEM, which was calculated by GraphPad Prism 8. *P* values were calculated by Student’s *t*-test using Excel. Nonlinear curve fitting and IC_50_ calculation was done with GraphPad Prism 8 using the inhibitor response three variable model or the exponential decay model. Images were prepared with Adobe Photoshop and assembled in Adobe Illustrator. All experiments presented were repeated at least twice independently except for the data from the Southern Research Institute, which was performed as fee-for-service in two duplicates. Data processing and reporting are adherent to the community standards.

## Supplementary information

Supplementary information

Supplementary information

Supplementary information

Supplementary information

Supplementary information

Supplementary information

Supplementary information

## Data Availability

All data, associated protocols, methods, and sources of materials can be accessed in the main text or supplementary information. The analysis code for drug synergy study is available at NCATS github. The mRNA sequencing data have been deposited to NCBI Sequence Read Archive. The accession ID is PRJNA645209.

## Supplementary information

**Supplementary Information** accompanies the paper at (10.1038/s41421-020-00222-5).
