# Supplementary material for: Heparan sulfate assists SARS-CoV-2 in cell entry and can be targeted by approved drugs in vitro
Source: Cell Discov. 2020 Nov 4;6:80. doi: 10.1038/s41421-020-00222-5 (PMC7610239; doi:10.1038/s41421-020-00222-5)
Supplement: Supplementary file 1 — Supplementary information [file 41421_2020_222_MOESM1_ESM.pdf]

List of supplementary materials:

Table S1 List of reagents used in the study

Figures S1-S5

Movies S1-S6

Supplementary table S1

Reagents and Materials	Source	Catalogue No.
pHrodo™ Red, succinimidyl ester (pHrodo™ Red, SE)	ThermoFisher Scientific	Cat# P36600
Banoxantrone dihydrochloride	Sigma	Cat# SML1854
Heparin sodium salt from porcine intestinal mucosa	Sigma	Cat# H3393
Heparan sulfate sodium salt	Biosynth Carbosynth	YH30121
Chondroitin sulfate sodium salt	Biosynth Carbosynth	YH04273
SARS-CoV-S and SARS-CoV2-S Pseudotyped particles	Codex Biosolutions (Gaithersburg, MD)	Contracted custom production
ATPLite	PerkinElmer	Cat# 6016736
Bright-Glo Luciferase kit	Promega	Cat# E2620
CellTiter-Glo cell viability kit	Promega	Cat# G7572
iScript™ Reverse Transcription Supermix for RT-qPCR	BioRad	Cat# 1708840
SsoAdvanced Universal SYBR Green Supermix	BioRad	Cat# 1725271
Imaging chamber	iBidi	Cat# 80426
TriPure reagent	Sigma	Cat# 11667157001
RNeasy MinElute Cleanup Kit	Qiagen	Cat# 74024
Latrunculin A	TOCRIS	Cat# 3973
NCGC00015693-09	Microsource	01503278
NCGC00015889-11	Microsource	01505622
NCGC00024857-02	BIOMOL	AC-304
NCGC00094226-07	SigmaAldrich	Lopac-P-0453
NCGC00094226-13	Selleck	S3026
NCGC00095196-06	Microsource	02300009
NCGC00164631-05	Sequoia	SRP01785s
NCGC00186034-01	SigmaAldrich	Lopac-K-1015
NCGC00186034-03	SIGMA	K1015
NCGC00249050-02	GVK	FFS-12-73-NCI-3078
Spike S1_S2	Sino Biological	40589-V27B-B
pLV-mCherry	A gift from Pantelis Tsoulfas (Addgene #36084)	
pcDNA3.1-SARS-CoV2-Spike	BEI resources	NR-52420
SARS-CoV-2 S antibody	GeneTex	Cat# GTX632604

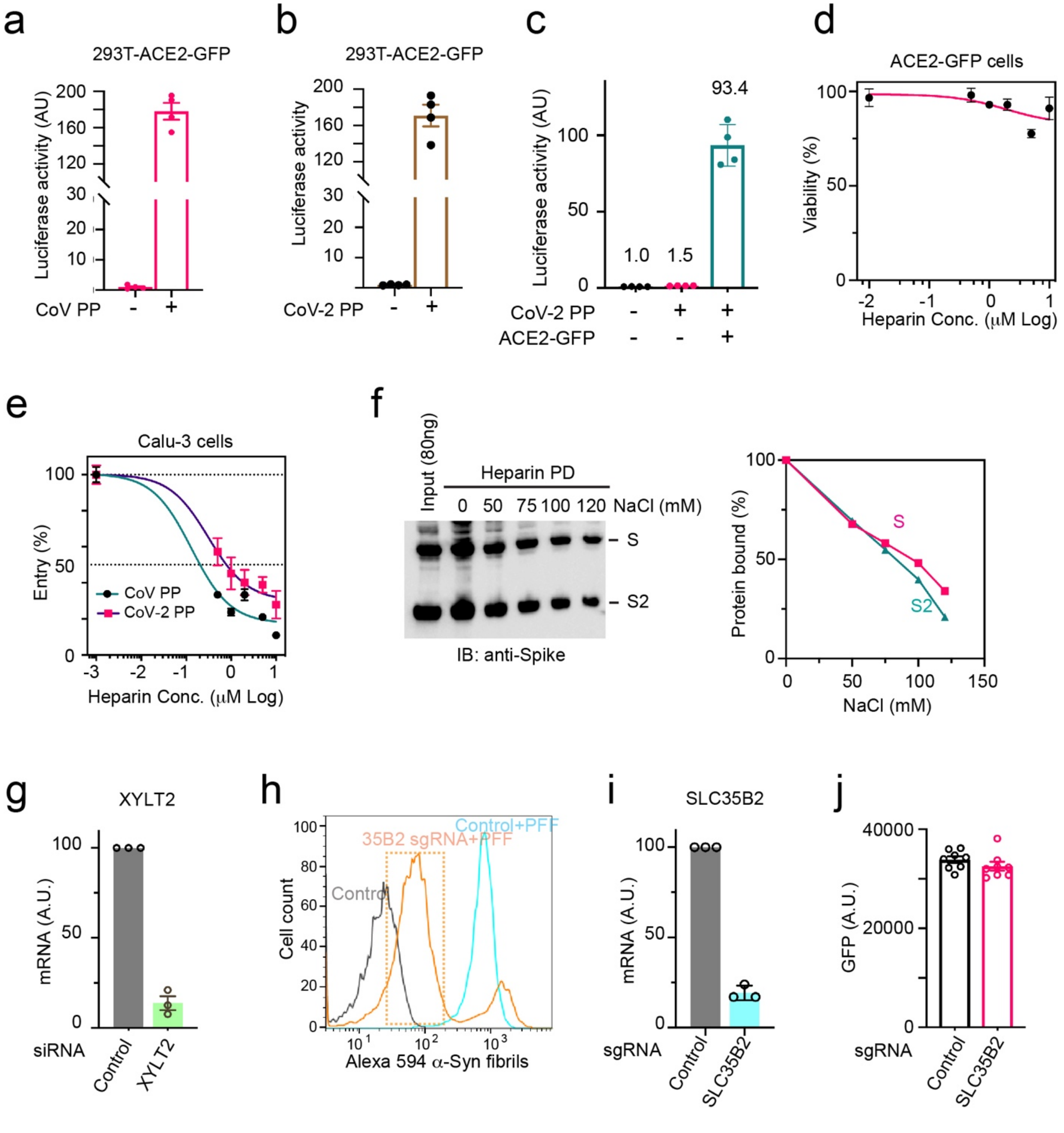

Supplementary Figure S1 Establishing a cell model to study Spike-dependent SARS coronavirus entry.

(**a, b**) The entry of SARS-CoV (a) or SARS-CoV-2 (b) PP in HEK293T ACE2-GFP cells. Uninfected cells or cells infected with the indicated PP for 24 h were analyzed for luciferase expression. The luciferase/GFP ratio was determined as an indicator of viral entry. Error bars indicate SEM. N=4.

(**c**) HEK293T cells or ACE2-GFP stable HEK293T cells were treated with SARS-CoV-2 PP as indicated. Luciferase expression was measured 48 post infection. Error bars indicate SEM. N=4.

(**d**) Cytotoxicity of heparin in HEK293T ACE2-GFP cells. Cell viability was measured 24 h after heparin treatment. Error bars indicate SEM. N=4.

(**e**) Heparin mitigates the entry of SARS-CoV and SARS-CoV-2 pseudoviral particles (PP) in Calu-3 cells. Calu-3 cells were transduced with SARS-CoV and SARS-CoV-2 PP in the presence of heparin as indicated. The luciferase levels were measured 48 h post-transduction. Error bars indicate SEM, N=4.

(**f**) Salt sensitive interaction of Spike with heparin. Spike (300 ng) or ACE2-mFC (300 ng) was incubated with heparin beads in the presence of salt as indicated. Proteins pulled down (PD) were analyzed by immunoblotting with anti-S2 antibodies. The graph shows the quantification of the experiment.

(**g**) Verification of *XYLT2* knockdown by qRT-PCR. A fraction of ACE2-GFP cells transfected with SMARTpooled *XYLT2* siRNAs or a control siRNA for 72 h were analyzed for *XYLT2* expression by qRT-PCR. The remaining cells were used in Figure 1f for viral entry and cell viability assay. Error bars indicate SEM, N=3 technical repeats.

(**h**) Generating ACE2-GFP cells deficient for *SLC35B2* (*35B2*). ACE2-GFP cells treated with *SLC35B2* sgRNA-expressing lentiviruses were incubated with Alex^{594}-labeled (400 nM) α-Syn

fibrils for 4h. α-Syn negative *SLC35B2* knockout (KO) cells (dashed box) were identified and collected by FACS.

(**i**) Verification of *SLC35B2* knockdown by qRT-PCR. A fraction of ACE2-GFP cells transfected with SMARTpooled *SLC35B2* siRNAs or a control siRNA for 72 h were analyzed for *SLC35B2* expression by qRT-PCR. The remaining cells were used for viral entry and cell viability assay in Figure 1g. Error bars indicate SEM, N=3 technical repeats.

(**j**) The knockout of *SCL35B2* does not affect ACE2-GFP expression. The ACE2-GFP level in cell extracts used in Figure 2c was determined by a fluorometer.

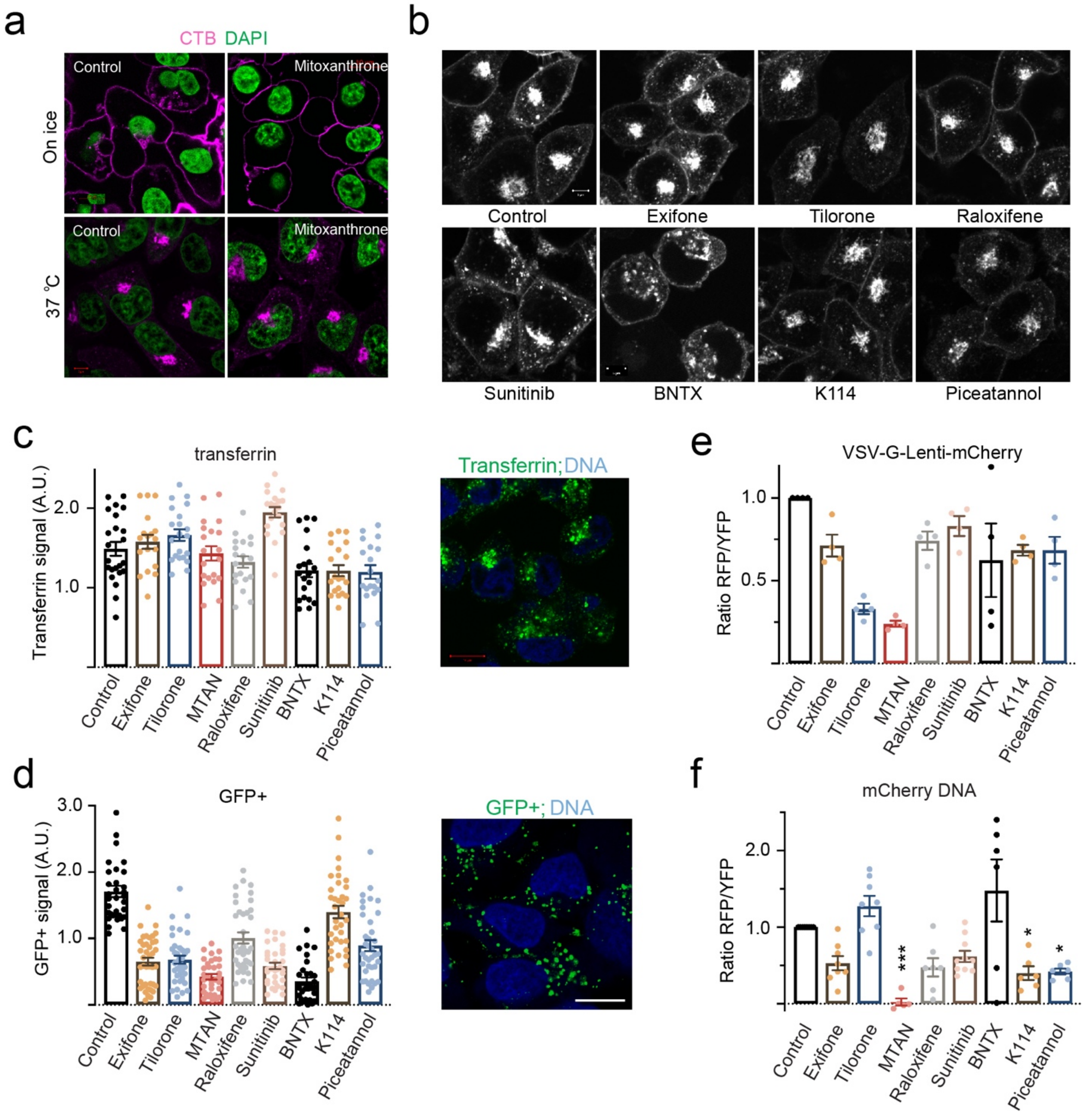

Supplementary Figure S2 Substrate specificity of the identified endocytosis inhibitors.

(a, b) Clathrin-independent endocytosis of cholera toxin B (CTB) chain is not affected by the identified drugs. (a) HEK2923T cells were treated with Mitoxantrone (5 μM) for 1h before incubation with 2 μg/ml Alexa[555]-labeled CTB on ice for 15min (cell surface binding) or at 37 °C

for 20min (uptake). Cells were fixed and stained with DAPI. Note that Mitoxantrone affects neither the binding of CTB to the cell surface nor its uptake. (b) Cells were treated with the indicated drugs at 10 μM (Sunitinib at 5 μM) for 1h before incubation with CTB at 37 °C for 20 min.

(**c**) The effect of drugs on the endocytosis of Transferrin. HEK293T cells were pretreated with the compounds for 30 min before incubation with Alexa^{488}-labeled Transferrin (50 μg/ml) at 37 °C for 4 h. Cells were fixed, stained with DAPI (blue), and imaged by confocal microscopy. The image shows an example of Transferrin uptake in control cells. The graph shows the quantification of internalized Transferrin signal in individual cells. A.U., arbitrary unit. Error bars indicate SEM.

(**d**) The effect of drugs on the endocytosis of GFP+. HEK293T cells treated with the indicated compounds for 30 min were incubated with 100 nM GFP+ for 4 h before heparin wash, fixation, and staining with Hoechst (Blue). The graph shows internalized GFP+ signals in individual cells. The image shows an example of GFP+ uptake in control cells. A.U. arbitrary units. Error bars indicate SEM. N=2.

(**e**) The effect of drugs on the entry of VSV-G-pseudotyped lentivirus bearing a mCherry reporter. HEK293T cells stably expressing YFP were treated with the inhibitors as in c, infected with VSV-G-Lenti-mCherry in the presence of the inhibitors for 6 h. Cells were then incubator in virus-free, inhibitor-free medium for 48 h before quantification of the mCherry /YFP ratio by a fluorometer. Error bars indicate SEM. N=4.

(f) The effect of drugs on the entry of plasmid DNA bearing a mCherry reporter. As in e, except that cells were transfected with a mCherry-bearing plasmid. Error bars indicate SEM. N=dot number. *, $p<0.05$, *** $p<0.001$ by unpaired student t-test.

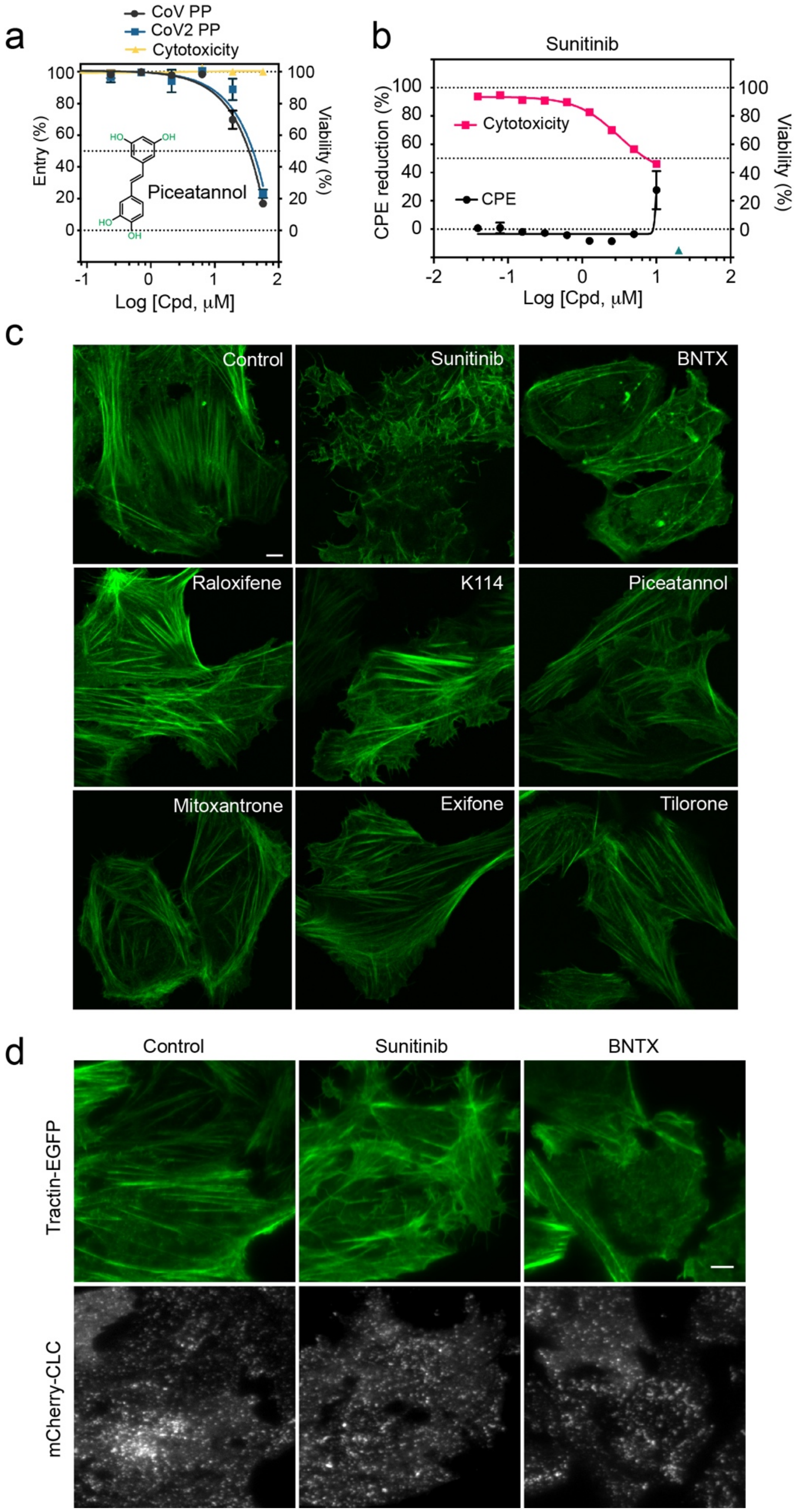
a
CoV PP
CoV2 PP
Cytotoxicity
Entry (%)
Viability (%)
Piceatannol
Log [Cpd, μM]
b
Sunitinib
CPE reduction (%)
Viability (%)
Cytotoxicity
CPE
Log [Cpd, μM]
c
Control
Sunitinib
BNTX
Raloxifene
K114
Piceatannol
Mitoxantrone
Exifone
Tilorone
d
Control
Sunitinib
BNTX
Tractin-EGFP
mCherry-CLC

Supplementary Figure S3 The effect of inhibitors on the actin cytoskeleton network.

(**a**) HEK293 ACE2-GFP cells were infected with SARS-CoV or SARS-CoV-2 PP in the presence of Piceatannol as indicated. Luciferase expression was determined 48 h post infection as an indicator of viral entry. Cells treated with the inhibitors without the virus were used to determine drug toxicity. Error bars indicate SEM, N=4.

(**b**) Sunitinib partially protects Vero E6 cells from SARS-CoV-2-induced cytopathic effect (CPE). Viability of Vero E6 cells was measured after treatment with the indicated drugs in the presence (black curve) or absence (red curve) of the SARS-CoV-2 virus for 72 h. Error bars indicate SEM, N=2.

(**c**) Confocal images of U2OS cells stably expressing Tractin-EGFP, which have been treated with the indicated inhibitors at 10 μM (Mitoxantrone and Sunitinib 5 μM) for 1h. Scale bar, 5 μm.

(**d**) TIRF images of drug-treated U2OS cells stably expressing Tractin-EGFP and mCherry-Clathrin light chain (CLC). CLC was used to focus on the plasma membrane. Scale bar, 5 μm.

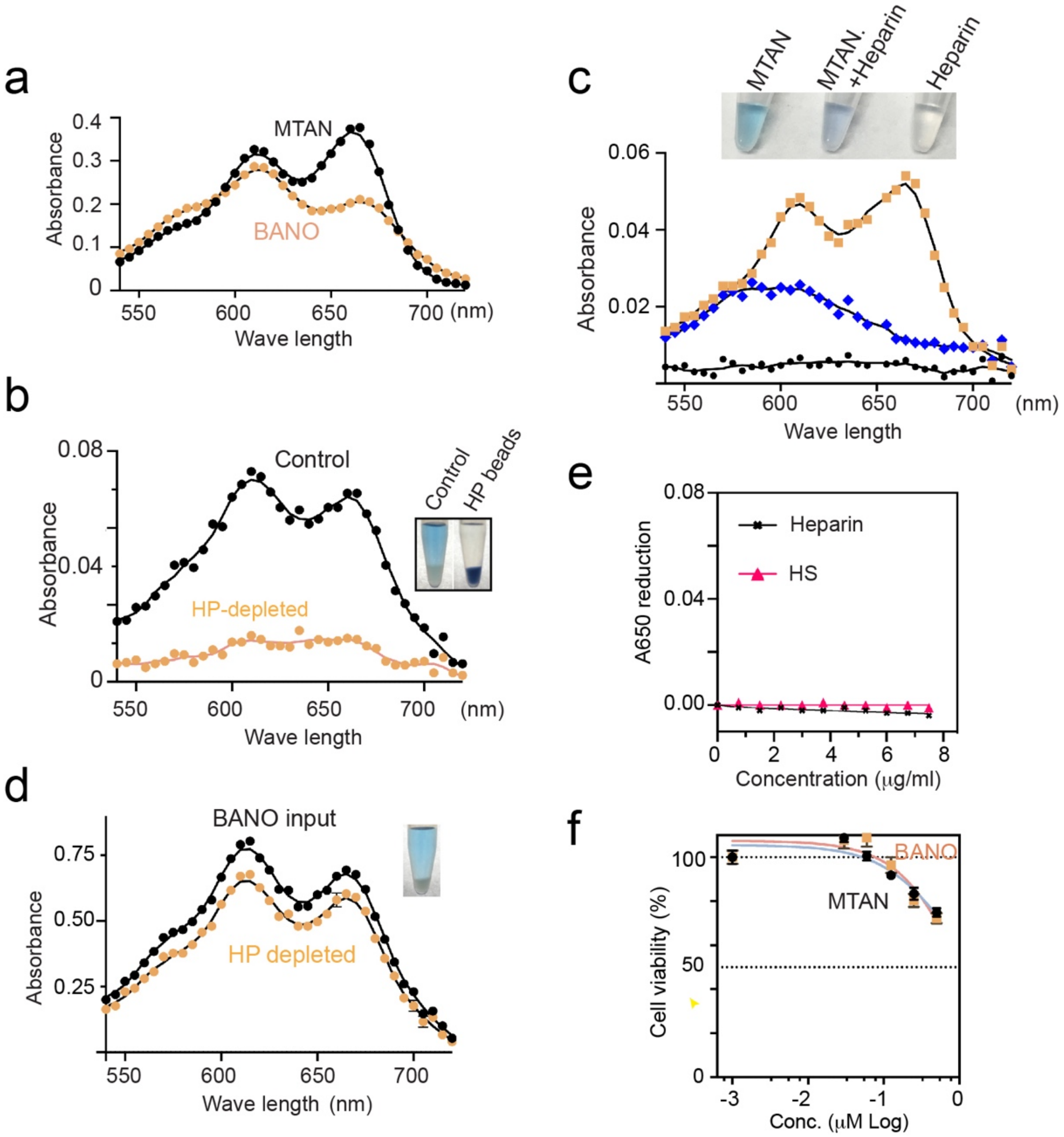

Supplementary Figure S4 Mitoxantrone but not Banoxantrone binds heparin.

(**a**) The absorption spectra of Mitoxantrone (MTAN) and the structurally related chemical Banoxantrone (BANO).

(**b**) Mitoxantrone binds heparin Sepharose. Mitoxantrone (50 μM) was incubated with either control Sepharose or heparin (HP)-coated Sepharose for 5 min. The picture shows the samples after centrifugation. The graph shows the absorbance spectra of the supernatant fractions.

(**c**) The interaction of heparin with Mitoxantrone changes its absorption spectrum. The absorption spectrum of Mitoxantrone (25 μM), heparin (25 μM), or Mitoxantrone+heparin (25 μM each) were determined by a NanoDrop spectrometer. The picture shows the color of the solutions.

(**d, e**) Banoxantrone (BANO) does not bind to heparin or HS. (d) Banoxantrone (50 μM) was incubated with heparin (HP)-coated Sepharose for 5 min. The picture shows the sample after centrifugation. The absorption spectrum of the HP-bead-depleted supernatant was measured together with a fraction of the input. (e) Banoxantrone (5 μM) was incubated with the indicated concentration of heparin or HS. A_{650} was measured. The changes in A_{650} after the addition of the oligosaccharides were plotted.

(f) The cytotoxicity profiles or Mitoxantrone and Banoxantrone in ACE2-GFP-expressing HEK293T cells. Error bars indicate SEM. N=3.

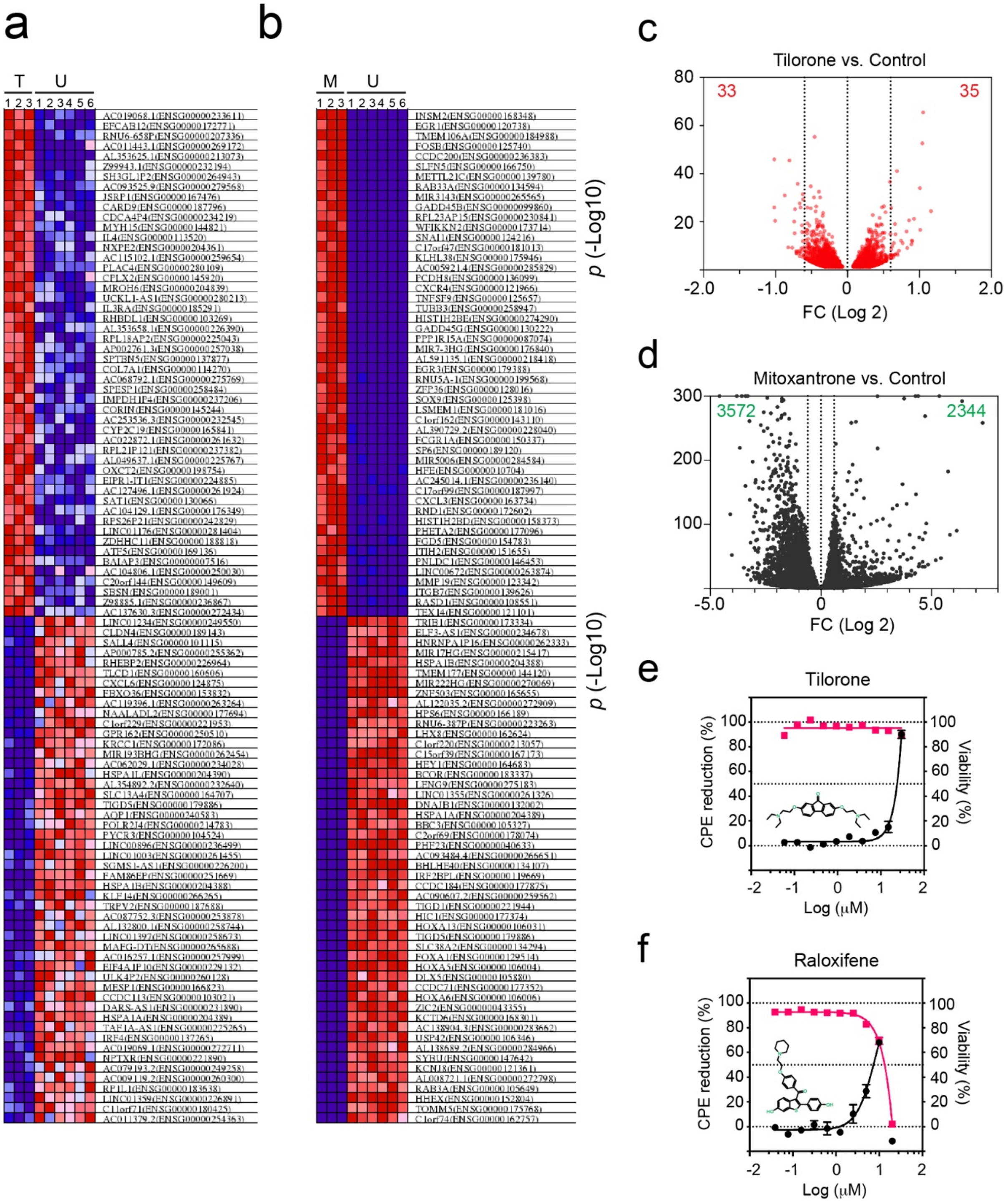

Supplementary Figure S5 Gene expression analyses of Tilorone- and Mitoxantrone-treated cells.

(**a**) A heat map summary of the most induced or down-regulated genes by Tilorone treatment (T, 10 μM 6 h) or in untreated (U) HEK293T cells.

(**b**) A heat map summary of the most induced or down-regulated genes by Mitoxantrone treatment (M, 5 μM 6 h) or in untreated (U) HEK293T cells.

(**c**) A volcano plot shows the genes affected by Tilorone. Note that both the number of genes (shown by the number) and the scale of the expression changes in Tilorone-treated cells are small.

(**d**) A volcano plot shows the genes affected by Mitoxantrone.

(**e**, **f**) The CPE inhibitory activity and cytotoxicity of Tilorone and Raloxifene. Vero E6 cells were treated with Tilorone (e) or Raloxifene (f) in the presence (CPE) or absence of wild-type SARS-CoV-2. Cell viability was measured 72 h after treatment.

Supplementary movies

Supplementary movie 1

A U2OS cell expressing Tractin-EGFP.

Supplementary movie 2

The cell shown in movie 1 was imaged after treatment with Sunitinib 5 μM for 15 min. Note the increased assembly of actin filaments causes new filopodia formation on the cell surface.

Supplementary movie 3

A Tractin-EGFP-expressing U2OS cell was imaged after treatment with Sunitinib 5 μM for 60 min.

Supplementary movie 4

The same Tractin-EGFP-expressing U2OS cell in movie 3 was imaged at a different confocal plan where the stress fibers are located.

Supplementary movie 5

A U2OS cell expressing Tractin-EGFP.

Supplementary movie 6

The cell shown in movie 5 was imaged after treatment with BNTX 10 μM for 5min.
